# *midline* represses Dpp signaling and target gene expression in *Drosophila* ventral leg development

**DOI:** 10.1242/bio.059206

**Published:** 2022-05-24

**Authors:** Lindsay A. Phillips, Markle L. Atienza, Jae-Ryeon Ryu, Pia C. Svendsen, Lynn K. Kelemen, William J. Brook

**Affiliations:** Alberta Children's Hospital Research Institute, Department of Biochemistry and Molecular Biology, Cumming School of Medicine, University of Calgary, 3330 Hospital Drive NW, Calgary, AB T2N 4N1, Canada

**Keywords:** Tissue patterning, Limb development, Dpp-signaling, T-box transcription factors

## Abstract

Ventral leg patterning in *Drosophila* is controlled by the expression of the redundant T-box Transcription factors *midline* (*mid*) and *H15*. Here, we show that *mid* represses the Dpp-activated gene *Daughters against decapentaplegic* (*Dad*) through a consensus T-box binding element (TBE) site in the minimal enhancer, Dad13. Mutating the Dad13 DNA sequence results in an increased and broadening of *Dad* expression. We also demonstrate that the engrailed-homology-1 domain of Mid is critical for regulating the levels of phospho-Mad, a transducer of Dpp-signaling. However, we find that *mid* does not affect all Dpp-target genes as we demonstrate that *brinker* (*brk*) expression is unresponsive to *mid*. This study further illuminates the interplay between mechanisms involved in determination of cellular fate and the varied roles of *mid*.

## Abstract (precis)

**Summary:** Ventral patterning is controlled in part by the T-box Transcription factor *midline* blocking Dpp signaling and Dpp-activated genes, though *midline* does not affect the Dpp-repressed gene *brk*.

## INTRODUCTION

Proper organization of tissue is crucial for maintaining the body plan of animals. This organization occurs during development when multiple factors cooperate to determine cell fate. In particular, the *Drosophila melanogaster* leg is organized in part by the interplay between signaling molecules and transcription factors. Dorsal and ventral fates in *Drosophila* legs are dependent on the action of morphogens and selector genes. The morphogens decapentaplegic [*dpp*, a bone morphogenetic protein (BMP) homolog] and *wingless* (*wg*, a fly Wnt) are induced by Hedgehog signaling. *Wg* is induced in the ventral domain and controls ventral fate through induction of the redundant Tbx20 class T-box transcription factor homologs *midline* (*mid*) and *H15*, that specify ventral fate (Svendsen et al., 2009). Dorsal fate is dictated by *dpp*, which is expressed at high levels in the dorsal domain and low levels in the ventral domain, though it has no role in ventral fate aside from joint formation (Held and Heup, 1996; Manjón et al., 2007). Dpp-signaling is mediated by transcription factors Mothers against dpp (Mad), a fly Smad1/5 activator and Medea (Med), a fly Co-Smad (Hudson et al., 1998; Kim et al., 1997).

*mid* and *H15* control ventral patterning being both necessary and sufficient to specify the fate in the ventral region of fly legs (Svendsen et al., 2009). They act as selector genes, transcription factors that specify cell fate for a particular developmental region, with expression restricted to the region in which they specify cell fate (Mann and Morata, 2000). The ventral specific expression of *mid* and *H15* is controlled through a combination of Wg activation and Dpp repression (Svendsen et al., 2009, 2015). When *mid* and *H15* function is lost in the ventral leg, tissues are transformed into dorsal while ectopic expression of *mid* or *H15* induces ventral fate in dorsal regions (Svendsen et al., 2009).

How does *mid* control ventral development? We have shown that one role of *mid* is to block Dpp signaling in the ventral domain (Svendsen et al., 2019). The distribution of phosphorylated Mad (pMad) follows the same pattern as *dpp,* with significant levels of staining in the dorsal domain and weaker staining in ventral region, indicating that the Dpp pathway is activated at lower levels in ventral cells. While Dpp does not contribute to ventral patterning, double-mutant analysis shows that the ventral to dorsal transformation of *mid H15* mutant clones in some regions of the leg is rescued to ventral phenotype if they are simultaneously blocked for Dpp signaling. This indicates an inhibitory effect of *mid* and *H15* on Dpp signaling that is necessary for ventral patterning. This is further demonstrated by the inhibition of pMad accumulation by *mid* (Svendsen et al., 2019). Additionally, *mid-*expressing clones repress the Dpp-target genes *Dad*, *Upd3,* and *mid* itself in an eh1-dependent manner, and using chromatin-immunoprecipitation (ChIP) assays, Mid has been shown to localize to enhancers for these genes (Svendsen et al., 2019).

Here, we study further the way *mid* antagonizes Dpp signaling, showing that it represses the regulation of *Dad*, a target gene activated by Dpp/pMad, but has no effect on *brk*, a gene repressed by Dpp/pMad. We show that Mid repression of *Dad* depends on a predicted T-box binding element (TBE) in the Dad13 enhancer, and that Mid-inhibition of Dpp-dependent pMad accumulation and tissue re-patterning depends on the eh1 repressor domain.

## RESULTS

### *mid* blocks Dpp activation of Dad13 reporter expression

**Fig. 1.**
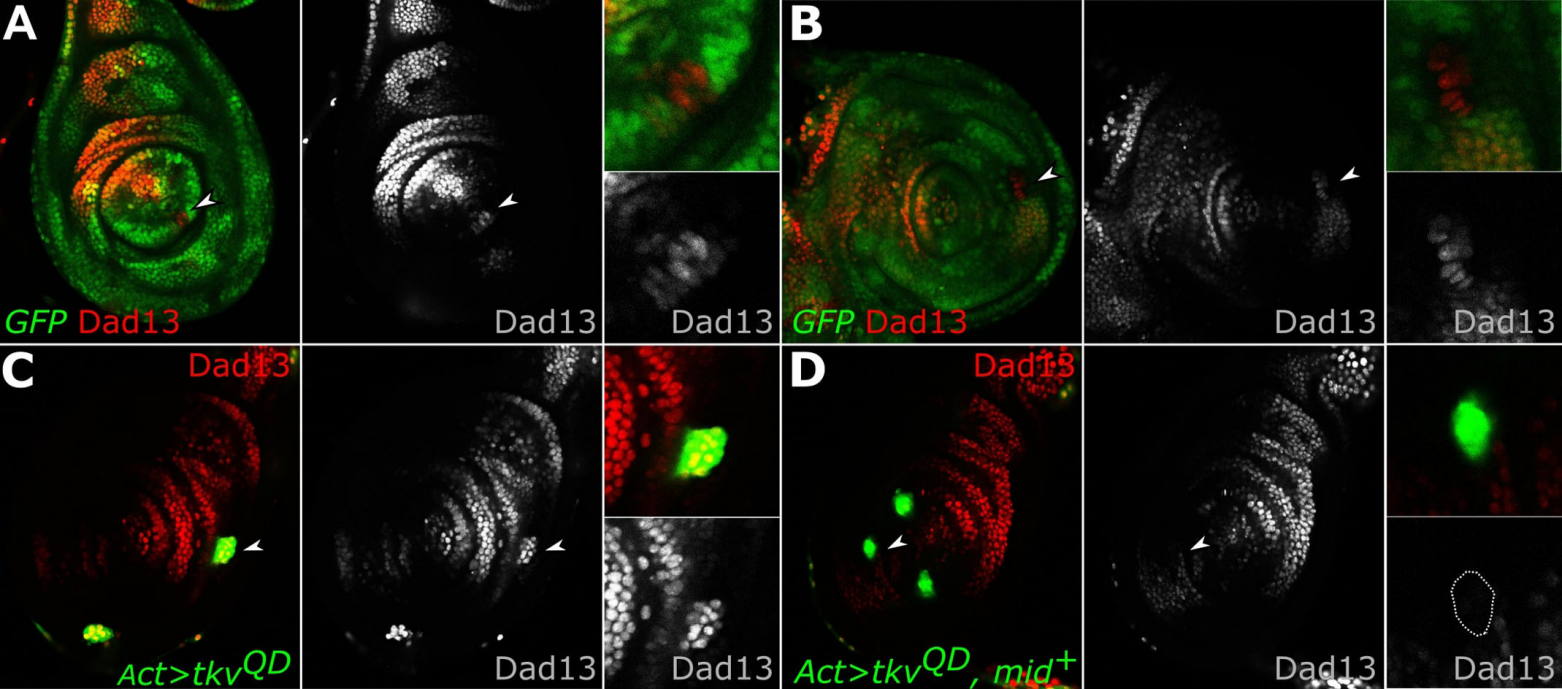
**Mid blocks Dpp activation of Dad13-driven expression.** (A-B) Third-instar leg discs expressing Dad13-nRFP (red, single channel) and loss-of-function *H15* and *mid* (lack of green) clones. Clones lacking *H15* and *mid* have (A) increased Dad13-nRFP reporter expression (white arrowhead, lower inset), or (B) expanded Dad13-nRFP reporter expression (white arrowhead, lower inset; *n*=7). (C) Discs with UAS-*tkv^QD^* gain-of-function clones (green) driven by *Ay*Gal4 driver showed ectopic Dad13-driven expression (red, single channel, lower inset; *n*=12), while (D) clones co-expressing UAS-*tkv^QD^* and UAS-*mid^+^* (green) did not induce ectopic Dad13-driven expression (red, single channel, lower inset clone outline; *n*=13). All imaginal discs in this report are orientated dorsal up, anterior left.

Our previous work showed that the eh1 repressor binding domain was required for Mid selector gene function. We also showed that Mid acted by antagonizing Dpp signaling, reducing the levels of pMad accumulation in the ventral domain of the fly leg (Svendsen et al., 2019). Here, we further investigate how *mid* affects Dpp-target gene expression. The Dpp-target genes *Dad* and *brk* are canonical examples of genes activated or repressed by Dpp signaling, respectively (Gao et al., 2005; Tsuneizumi et al., 1997). Since *mid* antagonizes Dpp signaling, we sought to understand how *mid* regulates both genes. We showed previously that an enhancer-trap reporter of *Dad* (*Dad*-lacZ) was weakly repressed by Mid in an eh1-dependent manner (Svendsen et al., 2019). Mid likely antagonizes Dpp-target genes through direct repression because Mid has been shown to bind to several enhancer fragments of *Dad* in ChIP assays (Svendsen et al., 2019). One of these fragments was a well-characterized 520 bp enhancer, Dad13. The Dad13-driven expression pattern is similar to that of *Dad-*lacZ, with strong expression in the dorsal domain and weaker expression in the ventral domain. However, the ventral Dad13-driven expression is weaker compared to wild-type *Dad*. We first confirmed that a Dad13 reporter was regulated by Mid. We investigated whether loss of *H15* and *mid* function would affect Dad13-driven expression by generating *H15 mid* loss-of-function clones via mitotic recombination in imaginal discs of second instar larvae. Dad13-driven expression was detected by RFP expression while clones null for *H15 mid* were marked by the absence of GFP. Ventrally located *H15 mid* loss-of-function clones showed either an increase of Dad13-driven expression or an expansion into the lateral region of the leg imaginal disc (Fig. 1A,B). Because the *mid* expression domain completely encompasses the ventral Dad13 domain (Fig. S1C), we induced ectopic Dpp-signaling in lateral regions of the imaginal disc in order to induce Dad13-reporter expression outside the *mid-*expression domain. We then introduced *mid* expression to assess the ability of Mid to repress Dad13. By using *Ay*Gal4, a construct that generates random clones expressing Gal4 under the control of the *actin5C* promoter, we induced clones marked by GFP expression that also expressed a constitutively active form of the Dpp receptor, *thickveins* (UAS-*tkv^QD^*) and/or expressed Mid. As expected, UAS-*tkv^QD^* expressing clones, which are constitutively activated for Dpp-signaling, had increased Dad13-nRFP reporter expression. However, co-expressing UAS-*mid^+^* and UAS-*tkv^QD^* blocked the ectopic activation of Dad13-nRFP in clones located throughout the disc (Fig. 1C,D). This indicates that the presence of Mid blocks the effects of Dpp-signaling on Dad13-driven expression. Together these results confirm that Mid does indeed regulate the Dpp-target gene *Dad* via its enhancer fragment, Dad13.

### The TBE in Dad13 is necessary for *mid* repression of *Dad*

**Fig. 2.**
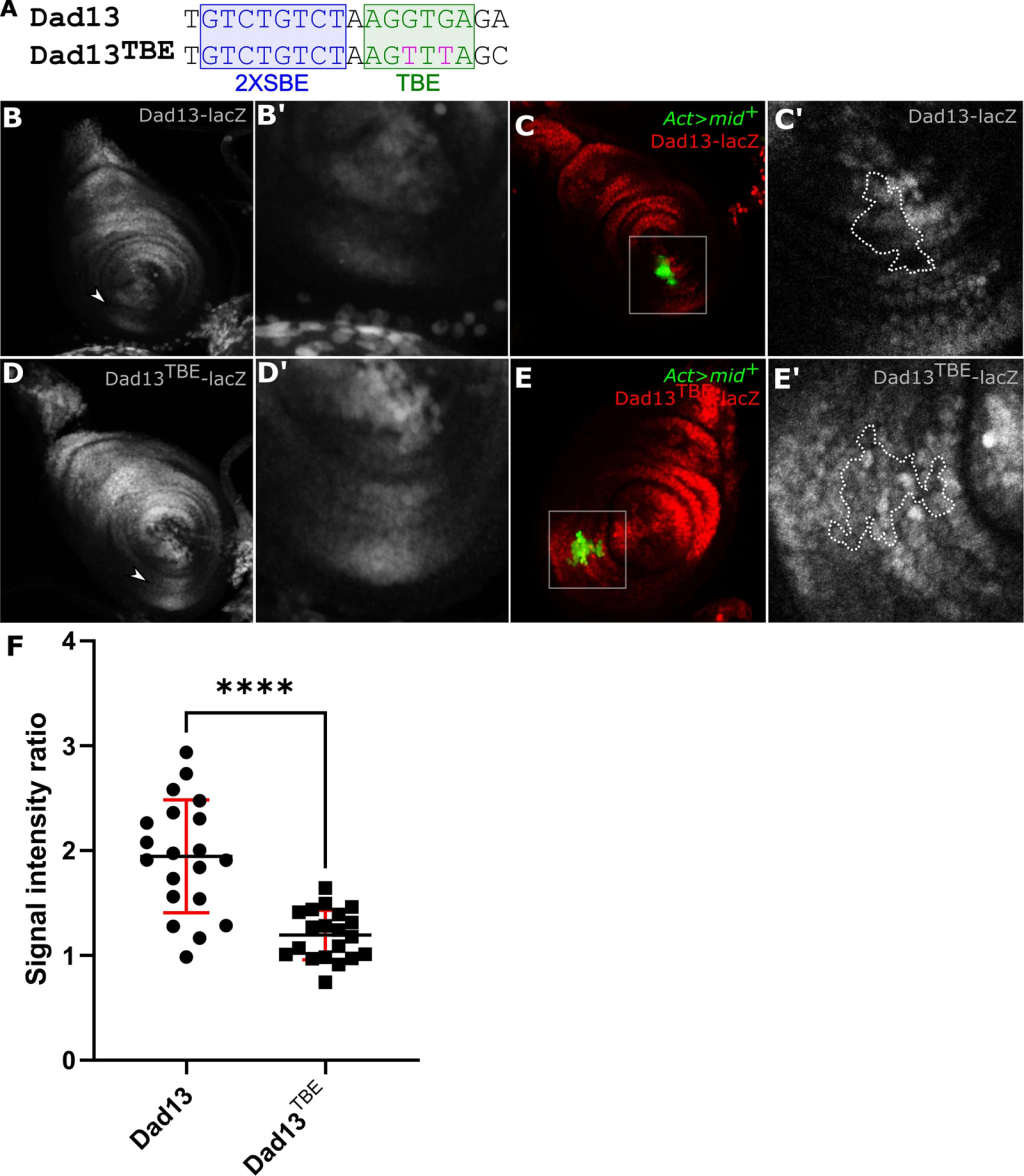
**Mid repressed Dad in part though the TBE.** (A) Dad13 enhancer fragment sequences, showing the 2x SBE sequence (blue) and the TBE sequence (green). Two G to T substitutions in the TBE were generated for the Dad13^TBE^ construct (fuchsia). (B) Dad13 expression was detected by lacZ with strong staining in the dorsal domain and weak staining in the ventral domain (arrowhead; *n*=37). (B′) Magnified image of ventral Dad13-lacZ reporter expression. (C,C′) UAS-*mid^+^* gain-of-function clones (GFP) partially repressed Dad13 expression (red channel, clone outline, C′; *n*=8). (D) Dad13^TBE^-lacZ reporter expression is broader and more intense in the ventral domain relative to Dad13 (arrowhead). Dorsal expression driven by Dad13^TBE^ is also stronger compared to Dad13 (*n*=35). (D′) Magnified image of ventral of Dad13^TBE^-lacZ reporter expression. (E,E′) Example of a UAS-*mid^+^* gain-of-function clone (GFP) which maintains normal levels of ventral Dad13^TBE^-lacZ reporter expression. No repression of Dad13^TBE^-lacZ reporter expression was seen compared to adjacent regions outside the clone (red channel, outline, E′; *n*=11). (F) Fluorescent signal intensities of Dad13-lacZ and Dad13^TBE^-lacZ for pairs of adjacent cells inside and outside of a *UAS-mid^+^* gain-of-function clone were measured and compared to create ratios of outside/inside. Five clones were measured for each Dad13 strain (Dad13-lacZ or Dad13^TBE^-lacZ) with two to five cell pairs being measured per *mid* clone. The overall mean ratio of cell pairs outside versus inside of a *UAS-mid^+^* gain-of-function clone in a Dad13-lacZ leg imaginal discs was 1.95. The overall mean ratio of cell pairs outside versus inside of a *UAS-mid^+^* gain-of-function clone in a Dad13^TBE^-lacZ leg imaginal discs was 1.19. Statistical analysis used the Unpaired *t*-test with two-tailed *P*-value. Bars representing the mean, s.d., and significance indicated, **** *P*-value≤0.0001.

To further investigate the role of *mid* in *Dad* regulation, we examined how Mid influences Dad13-reporter expression. The Dad13 enhancer contains multiple binding sequences for Mad that are responsible for driving Dad13 expression (Weiss et al., 2010). Dad13 also contains tandem Smad binding element (SBE) sequences (GTCTGTCT) that have a minor role in activating Dad13-reporters in embryos but which have not been tested in leg imaginal discs (Weiss et al., 2010). Adjacent to the SBE sites and separated by a single nucleotide is a consensus T-box binding element (TBE) (AGGTGA) similar to the consensus binding site for Mid (Najand et al., 2012). To test whether the TBE is necessary for Mid regulation of Dad13, we mutated the Dad1-TBE site (Dad13^TBE^) and tested expression with a lacZ reporter (Fig. 2A). Dad13^TBE^-lacZ reporter expression was stronger and broader in the ventral domain compared to the Dad13-lacZ control (Fig. 2B,D). This clearly indicates that the TBE is required for repression of *Dad.* Furthermore, the Dad13^TBE^-driven expression in dorsal regions outside the *mid* expression domain was more intense than Dad13-driven expression, suggesting that factors expressed outside the ventral domain may also regulate *Dad* through the TBE site. The proximity of the activating SBE next to the potential Mid-binding element suggested a model in which the activation of Dad13 by Dpp may be blocked by Mid or other factors interfering with the SBE site. However, mutating the SBE (Dad13^SBE^) had no observable effect on Dad13-driven expression and mutating both the TBE and SBE (Dad13^SBE+TBE^) produced effects on reporter expression that were similar to mutating the TBE alone (Fig. S3). Thus, the SBE site does not appreciably affect Dad13-driven expression in the leg imaginal disc and the TBE site must affect Dad13 enhancer through another mechanism.

To test whether Mid could still regulate Dad13^TBE^-driven expression, we generated UAS-*mid^+^* gain-of-function clones and measured Dad13^TBE^lacZ expression. Consistent with our previous work (Svendsen et al., 2019), Dad13-reporter expression was repressed in most *mid-*expressing clones in the ventral domain (7/8) (Fig. 2C,C′). In contrast, few *mid-*expressing clones in the ventral domain of discs a decreased Dad13^TBE^- driven expression (1/11 clones; Fig. 2E,E′). To further validate that *mid-*expressing clones had weaker effects on Dad13^TBE^-reporter expression, we measured the expression in pairs of adjacent cells located inside and outside of the *mid*-expressing clone. The ratio of reporter expression of the outside cell divided by the reporter expression of the inside cell was 1.95 for Dad13 and 1.19 for Dad13^TBE^ (Fig. 2F). These results suggest that the wild-type TBE in the Dad13 enhancer fragment is an essential element for the *mid-*mediated repression of *Dad*.

### *mid* does not substantially affect Dpp repression of *brk*

**Fig. 3.**
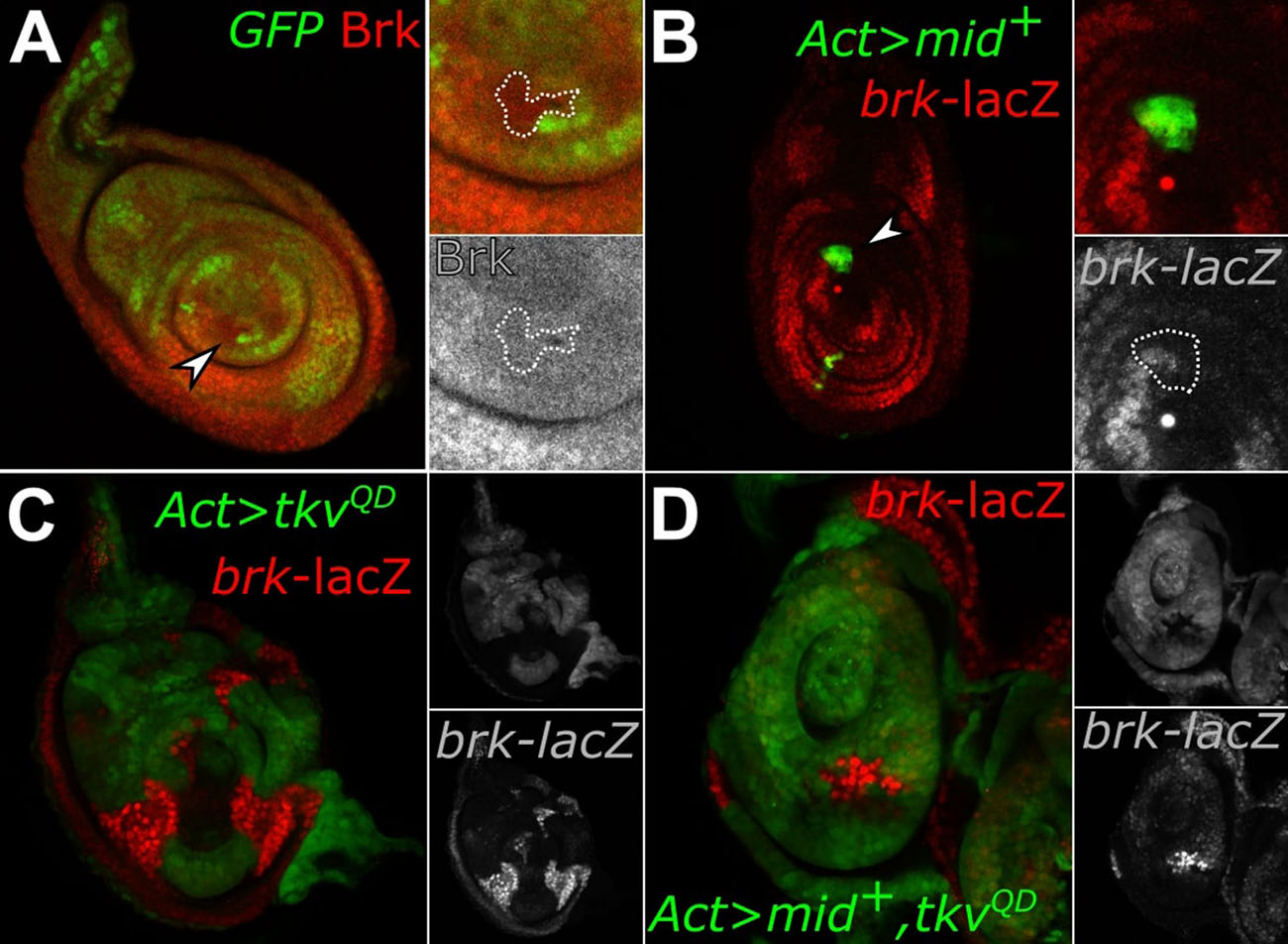
**Mid does not affect *brk* expression.** (A) *H15 mid* loss-of-function (lack of green, arrowhead) clones have no effect on Brk expression, as detected by a Brk antibody (red, lower inset clone outline; *n*=41). (B) Discs expressing *Ay*Gal4 UAS-*mid^+^* gain-of-function clones (green) do not affect *brk*-lacZ expression, as detected by anti-β-Galactosidase staining (red, lower inset clone outline; *n*=34). (C) Clones expressing UAS-*tkv^QD^* (green, top inset) have a dramatic repressive effect on *brk*-lacZ levels, with only residual *brk-*lacZ expression remaining (red, lower inset; *n*=7). (D) Clones co-expressing UAS-*tkv^QD^* and UAS-*mid^+^* (green, top inset) also greatly reduce *brk*-lacZ expression, with only slightly higher remaining *brk-*lacZ expression (red, lower inset; *n*=7).

*brk* is negatively regulated by Dpp signaling. Unlike *Dad*, which is activated by a complex of pMad and Med, *brk* is repressed by a complex of pMad and Med binding along with a third protein, Schnurri, through a repressive binding element (Hamaratoglu et al., 2014; Marty et al., 2000; Saller and Bienz, 2001; Weiss et al., 2010). In leg imaginal discs, high levels of *brk* and pMad expression are reciprocal, demonstrating their antagonistic activities (Müller et al., 2003; Fig. S1). We found that neither *H15 mid* loss-of-function clones nor *mid^+^*-expressing gain-of-function clones had any effect on *brk* expression. In loss-of-function experiments, endogenous *brk* expression was detected by a *brk* antibody and remained unchanged in ventrally located clones lacking *H15 mid* (Fig. 3A, insets). Moreover, *mid^+^* gain-of-function clones marked by GFP (UAS-*mid*^+^) and located throughout the imaginal disc did not affect *brk*-lacZ expression (Fig. 3B, insets). Because Dpp-signaling represses *brk* and *mid* represses *Dpp-*signaling, we tested if ectopic expression of Dpp signaling with *mid* would increase or otherwise affect *brk* expression. As expected, gain-of-function clones expressing UAS-*tkv^QD^* gain-of-function strongly repressed *brk-lacZ* reporter expression leaving only residual expression (Fig. 3C, insets). However, clones co-expressing UAS-*mid*^+^ with UAS-*tkv^QD^* had little influence on the *tkv^QD^* gain-of-function effect on *brk-*reporter expression, which remained almost as strongly repressed (Fig. 3D, insets). This was surprising given that clones co-expressing UAS-*mid^+^* and UAS-*tkv^QD^* have markedly reduced pMad staining compared to clones expressing UAS-*tkv^QD^* alone (Fig. S2; Svendsen et al., 2019). This suggests that clones co-expressing UAS-*mid^+^* and UAS-*tkv^QD^* have sufficient residual pMad activation to repress *brk* despite UAS-*mid^+^* expression. Taken together, these results suggest that *mid* does not regulate *brk* and thus *mid* does not affect all Dpp-target genes.

### Mid antagonizes Dpp signaling in an eh1-dependent manner

**Fig. 4.**
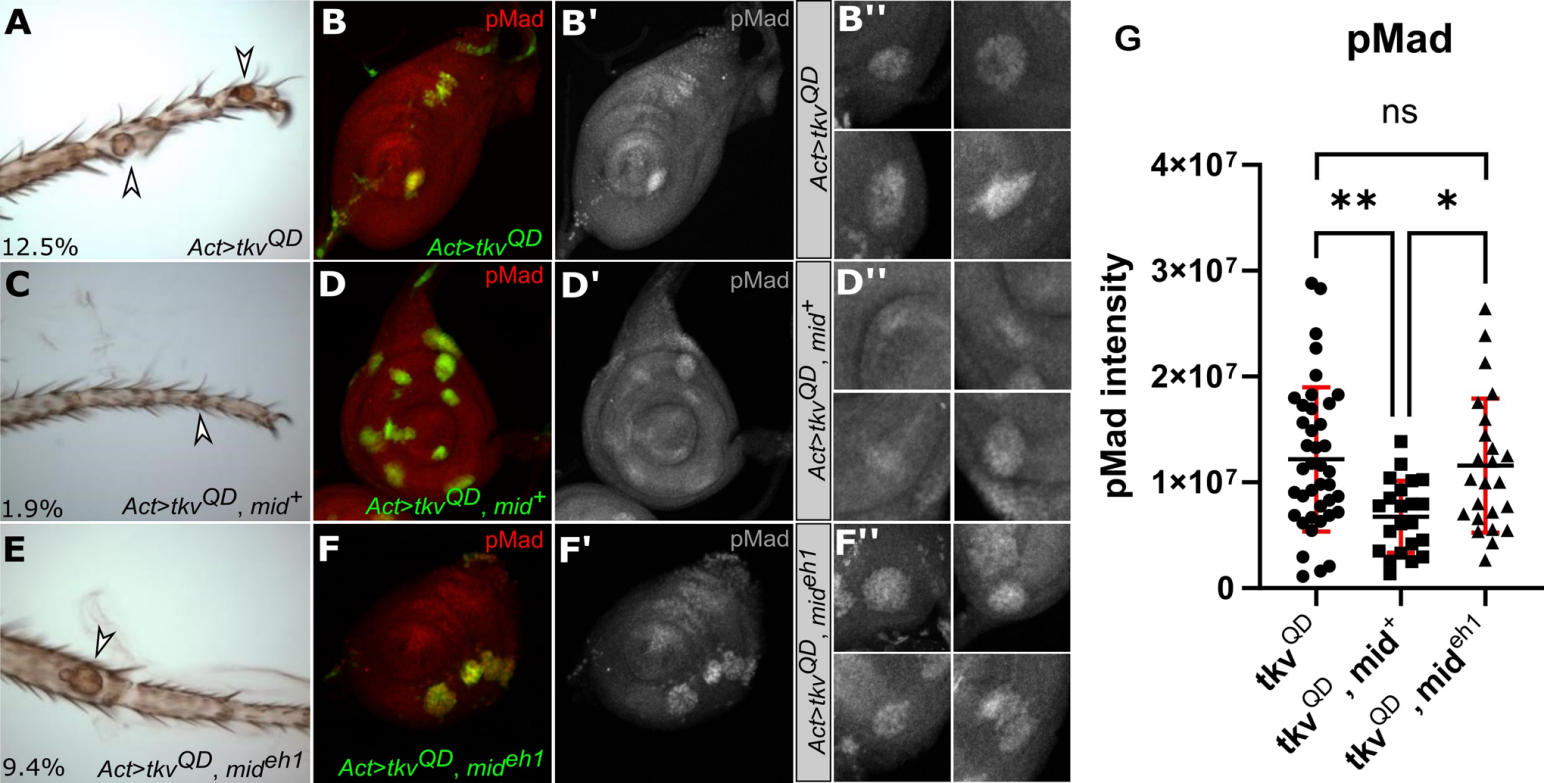
**The eh1 domain is involved in Dpp repression.** (A) *Ay*Gal4 gain-of-function clones expressing UAS-*tkv^QD^* result in outgrowths and dorsal transformation (arrowhead), 12.5% of legs had ectopic outgrowths under these conditions (*n*=181). (C) When UAS-*tkv^QD^* was co-expressed with UAS-*mid^+^-*Flag, the outgrowths were less severe (arrowhead) indicating suppression of the *tkv* gain-of-function phenotype. The effect was also less frequent with only 1.9% of legs having ectopic outgrowths (*n*=309). (E) Clones co-expressing UAS-*tkv^QD^* and UAS-*mid^eh1^-*Flag had deformities (arrowhead) similar to clones expressing UAS-*tkv^QD^* alone, where 9.4% of legs had ectopic outgrowths (*n*=106). The clones scored in adult cuticles in panels A, C and E are not marked in these experiments but resemble the effect of marked *tkv^QD^* clones in other experiments (data not shown). pMad staining (red, greyscale single channel) was upregulated in clones (green) expressing UAS-*tkv^QD^* (B,B′,B″; *n*=40) and clones co-expressing UAS-*tkv^QD^* and UAS-*mid^eh1^-*Flag (green) (F,F′,F″; *n*=24). When UAS-*mid^+^-*Flag was expressed in clones (green) along with UAS-*tkv^QD^*, pMad staining was less elevated (D,D′,D″; *n*=22). (G) The difference in pMad levels staining between UAS*-tkv^QD^* clones and UAS*-tkv^QD^,* UAS-*mid^eh1^-*Flag was not significant. However, clones expressing UAS*-tkv^QD^,* UAS-*mid^+^-*Flag had significantly lower pMad compared to the other two conditions. The mean values were UAS*-tkv^QD^* 1.22×10^7^, UAS*-tkv^QD^,* UAS-*mid^+^-*Flag 6.73×10^6^ and UAS*-tkv^QD^,* UAS-*mid^eh1^-*Flag 1.16×10^7^. Statistical analysis used the Tukey's multiple comparisons test, with bars representing the mean, s.d., and significance indicated, *P-value≤0.05 and **P-value≤0.01.

Mid acts as a repressor through its engrailed-homology-1 (eh1) domain, which recruits the co-repressor *groucho* (*gro*) (Formaz-Preston et al., 2012). The eh1 domain is essential for Mid-mediated repression of genes in the ventral leg including the Dpp-target gene *Dad* (Svendsen et al., 2019). Here we asked if the eh1 is also necessary to inhibit the effects of ectopic Dpp signaling, including phenotypic defects and pMad accumulation. We generated gain-of-function UAS-*tkv^QD^* clones in developing imaginal discs in second instar larvae. In adult legs, these clones gave rise to rounded outgrowths characteristic of ectopic Dpp-signaling (Fig. 4A; Svendsen et al., 2019). Next, we co-expressed UAS-*tkv^QD^* and a Flag-tagged *mid* (UAS-*mid^+^-*Flag) in adult legs. These clones induced fewer defects, which were less severe with generally smaller outgrowths (Fig. 4C). However, when we co-expressed UAS-*tkv^QD^* with UAS-*mid^eh1^-*Flag, a transgene in which the eh1 domain is mutated and has reduced Gro-binding (Formaz-Preston et al., 2012), the adult legs (Fig. 4E) displayed outgrowths similar in size and severity to clones expressing UAS-*tkv^QD^* alone (Fig. 4A). Overall, more defects were detected in legs containing either UAS-*tkv^QD^* or UAS-*tkv^QD^* and UAS-*mid^eh1^-*Flag clones as compared to legs expressing UAS-*tkv^QD^* and UAS-*mid^+^-*Flag clones.

In addition to the different patterning defects induced in adult legs, we also saw changes in the pMad accumulation in leg imaginal discs. Clones in third-instar discs expressing gain-of-function UAS-*tkv^QD^* have increased pMad staining (Fig. 4B,B′,B″). Similar levels of pMad were detected in clones co-expressing UAS-*tkv^QD^* and UAS-*mid^eh1^-*Flag (Fig. 4F,F′,F″). In comparison, clones expressing the UAS-*mid^+^-*Flag and UAS-*tkv^QD^* had lower levels of pMad staining relative to the other two genotypes (Fig. 4D,D′,D″,G). We note that the suppression of pMad by Flag-tagged UAS-*mid^+^-*Flag is less pronounced than the suppression by untagged UAS-*mid^+^* (Fig. S2; Svendsen et al., 2019). This is consistent with our observation that all Flag-tagged Mid gain-of-function phenotypes are weaker compared to untagged Mid (Formaz-Preston et al., 2012; Svendsen et al., 2019). However, the UAS-*mid^+^-*Flag is able to rescue *mid* mutants, and the UAS-*mid^+^-*Flag and UAS-*mid^eh1^-*Flag transgenes are well matched for expression levels, with UAS-*mid^eh1^-*Flag expressed approximately twofold higher than UAS-*mid^+^-*Flag (Formaz-Preston et al., 2012; Svendsen et al., 2019). Thus, despite being expressed at a higher level than the UAS-*mid^+^-*Flag strain, the UAS-*mid^eh1^-*Flag expressing clones have much weaker effects on pMad levels. Together, these results suggest that the eh1 domain is implicated in both of Mid's repressive roles: direct repression of Dpp-target genes and interference with the Dpp-signaling cascade.

## DISCUSSION

In this study, we investigated how the ventral selector gene *mid* antagonizes Dpp signaling. Specifically, we showed that *mid,* which antagonizes Dpp signaling and pMad accumulation, represses the Dpp-activated gene *Dad* but has minimal effects on the regulation of the Dpp-repressed gene *brk*. Furthermore, *mid* antagonizes dorsal fate by repressing *Dad* and by inhibiting Dpp signaling induced pMad accumulation via the eh1 domain.

We showed previously that Mid localizes to several Dad enhancers, including the *Dad13* enhancer, and represses a *Dad* enhancer trap in an eh1*-*dependent manner (Svendsen et al., 2019; unpublished data). Here we show that *mid* regulates Dad13-driven expression through a TBE site. Ventral Dad13-driven expression is increased and expanded in *mid* loss-of-function, while ectopic expression of *mid* blocks Dad13-reporter expression. Mutating the TBE in the Dad13 enhancer fragment increased Dad13 expression levels and expanded the ventral domain of expression compared with controls. Furthermore, the TBE mutation rendered the construct less sensitive to *mid^+^* gain-of-function. The localization of Mid to the Dad13 enhancer by ChIP (Svendsen et al., 2019) and the genetic results suggesting that the TBE is required for Mid repression of Dad13-expression supports direct repression of *Dad* by Mid through the TBE.

However, a surprising result in light of this proposal was that the dorsal Dad13^TBE^-driven expression outside the *mid* expression domain was also increased compared to Dad13. This suggests that the TBE sequence binds factors in dorsal cells to control the activity of the Dad13 enhancer. Recent work on *Tc-omb*, the *optomotor-blind* (*omb*) homolog in the red flour beetle, *Tribolium castaneum,* suggests one possible explanation. Like the *Drosophila* T-box factor *omb*, *Tc-omb* is expressed in the dorsal region of developing beetle legs and is required for dorsal patterning. Loss of function of dorsal *Tc-omb* results in increased pSmad levels in dorsal cells (Pechmann and Prpic, 2022). This is analogous to the increase in ventral pMad levels we find in *mid H15* loss of function (Svendsen et al., 2019). Although it is not known if *Tc-omb* loss of function also results in the increase of Dpp target genes in *T. castaneum*, it is interesting to speculate that perhaps dorsal T-boxes like *omb* play parallel roles to *mid* and *H15*, dorsal Dpp signaling and gene expression.

A second finding is that Mid suppresses pMad accumulation in a manner that is dependent on the eh1 domain. Mid mutants in which eh1 is compromised are unable to suppress Dpp gain-of-function effects. When co-expressed with Dpp-signaling overexpression in imaginal discs, clones of UAS-*mid^eh1^-*Flag maintain strong pMad staining and adult cuticles display outgrowths and deformities that resemble the effects of Dpp-signaling overexpression alone. Conversely, clones activated for Dpp signaling that express wild-type *mid* (UAS-*mid^+^-*Flag) have decreased pMad levels in imaginal discs, while adult legs have fewer and milder defects. This demonstrates that in addition to blocking Dpp-target genes such as *Dad*, *mid* represses genes that act to increase pMad levels and Dpp signaling. What these repressed target genes may be remains a subject for further study.

Our results showing that *brk* expression was not affected by *mid* were surprising to us because *brk* is a sensitive readout of Dpp signaling and because *mid* alters pMad levels. However, *mid* loss-of-function clones, which increase pMad levels, do not affect *brk* expression, even though *brk* is regulated by Dpp-signaling in ventral *mid*-expressing cells. It seems likely that *brk* is simply not sensitive to the modulations in pMad seen in *mid/H15* mutant clones. Additionally, *mid* expression was unable to substantially reverse the repression of *brk* by Dpp gain-of-function, despite *mid* dramatically decreasing pMad accumulation in this genetic background, suggesting that *brk* repression is sensitive to repression at low thresholds of Dpp signaling. This suggests that, overall, *mid* and *H15* do not contribute to the regulation of *brk*.

The lack of interaction between *mid* and *brk* in this study is consistent with previous work, where it was reported that the expression of the ventral genes *H15* and *wg*, and the dorsal genes *omb* and *dpp*, were normal in *brk* mutant leg discs, indicating that *brk* does not participate in the formation of the DV axis (Estella and Mann, 2008). Instead, *brk* functions to form the proximo-distal (PD) axis by antagonizing the Wg target genes *Distalless* (*Dll*) and *dachshund* (*dac*), helping to establish their expression in the PD axis. This leads to a model in which Dpp induces the PD and DV leg axes through two separate modes: Dpp inhibits *brk* repression of Wg targets to establish the PD axis and antagonizes Wg targets in ventral development through pMad/Med/Shn mediated repression (Estella and Mann, 2008). Thus, *mid* and *brk* may play parallel roles repressing Dpp-target genes in the D/V and P/D axes of the leg imaginal disc, respectively.

## MATERIALS AND METHODS

### Fly stocks and constructs

All flies were maintained on standard media containing cornmeal, agar, yeast, glucose, and water (Deliu et al., 2017) and housed at between 18^°^C-25^°^C. Stocks UAS-*tkv^QD^*, *brk-lacZ,* and *Dad*-lacZ were obtained from Bloomington Indiana Stock Center. *brk*-lacZ (BM315) was a gift from Dr. Konrad Basler, University of Zurich (Müller et al., 2003). H15^X4^ mid^1a5^, UAS-*mid^V5^*, (Svendsen et al., 2009) and UAS-*mid2.12* (Buescher et al., 2004) were generated previously.

### UAS-mid strains

In this study, *mid^+^* may refer to either UAS-*mid^V5^* or UAS-*mid2.12* and both lines have very similar levels of *mid^+^* activity*.* For quantitative comparisons of *mid^+^* and mutant *mid^eh1^*, previously generated strains with Flag-tagged constructs, UAS-*mid^eh1^-*Flag and UAS-*mid^+^-*Flag, were used (Formaz-Preston et al., 2012). The relative expression levels of these lines are such that *mid^eh1^* is expressed at roughly two-fold higher levels than *mid^+^* (Formaz-Preston et al., 2012; Svendsen et al., 2019). *UAS-mid^+^* strains have generally stronger effects than UAS-*mid^+^-*Flag in gain-of-function experiments, although both *UAS-mid^+^* and UAS-*mid^+^-*Flag are able to rescue *mid H15* loss-of-function (Formaz-Preston et al., 2012; Svendsen et al., 2019)

### Dad13 reporter strains

Dad13 constructs (Dad13, Dad13^TBE^, Dad13^SBE^, and Dad13^SBE+TBE^) were generated for this study using the pGL3basic-hsp70-dad13 (331) plasmid gifted by Dr. Giorgos Pyrowolakis, University of Freiburg, and mutations were made with an adapted splice protocol (Warrens et al., 1997) in a placZ-2.attB vector. All transgenes were inserted into P{CaryP}attP2 located at 68A4. The Dad13nRFP strain was a gift from Dr. Doug Allan, University of British Columbia.

### Loss-of-function and gain-of-function genetic mosaics

Heat shocking larvae 48-72 h after egg laying activates heat-shock-inducible hs-FLP, which is implemented in both gain-of-function and loss-of-function clonal experiments. In gain-of-function experiments, the combination of an *Ay*Gal4 construct with a UAS-linked gene allows for generation of ectopic clones which are labeled with GFP (Ito et al., 1997). Inducing the hs-FLP in loss-of-function experiments catalyzes mitotic recombination between FRT sites of homologous chromosomes (Xu and Rubin, 1993). This gives rise to two daughter cells homozygous for different genotypes, mutant and wild type. The consequent clones have wild-type cells marked with GFP, whereas loss-of-function cells lack GFP.

### Reporter constructs, immunohistochemistry and imaging

The expression of *Dad*-lacZ (Tsuneizumi et al., 1997), *brk*-lacZ (Müller et al., 2003), and Dad13 constructs were detected using mouse-anti-β-galactosidase (1:1000, Promega catalogue number Z3781) and rabbit-anti-β-galactosidase (1:1000, Cell Signaling Technology, catalogue number 27198). *Brk* was also detected with rat anti-Brk (1:100, a gift from Gines Morata, Universidad Autonoma de Madrid; Moreno et al., 2002). Dad13 was visualized by RFP expression. pMad was detected with the rabbit-anti-pSmad1/5/9 antibody (1:200, Cell Signaling Technology, catalogue numbers S463/465/ S465/467, D5B10 13820S). Secondary antibodies used were Alexa-fluor 546 and 488 against rabbit, rat, or mouse (1:500, molecular probes A11029, lot 504513, A11094, lot 47207A, A11030, lot 517979, A11035, lot 584959, A11081, lot 58080A). Imaginal discs were imaged on a Zeiss LSM 700 confocal microscope using the 20x objective lens with ZEN 2.3 SP1 FP3 Black edition software. All compared genotypes were imaged at the same acquisition settings to maintain intensities. Adult tissues were visualized on a Leica MPS60 compound microscope with a 10x or 20x objective and QCapture_x64 software. Z-stack CZI files generated on the Zeiss LSM 700 Axio Observer confocal microscope were processed and analyzed with ImageJ software (version 1.53c, Java 1.8.0_172, 64-bit) and saved as JPEG files. Some files were then further analyzed to determine fluorescence intensity in according to published methods (McCloy et al., 2014). Images were imported into INKScape (version 1.0.2-2, e86c870879, 2021-01-15) to generate figures for this paper. Graphs were generated and statistical analysis performed in GraphPad Prism (version 9.2.0) using either unpaired *t*-test (Figure 2) or ordinary one-way analysis of variance (ANOVA) (Figure 4) with Tukey's multiple comparisons test.

## Supplementary Material

Supplementary information
