# Supplementary material for: midline represses Dpp signaling and target gene expression in Drosophila ventral leg development
Source: Biol Open. 2022 May 24;11(5):bio059206. doi: 10.1242/bio.059206 (PMC9167623; doi:10.1242/bio.059206)
Supplement: Supplementary information [file biolopen-11-059206-s1.pdf]

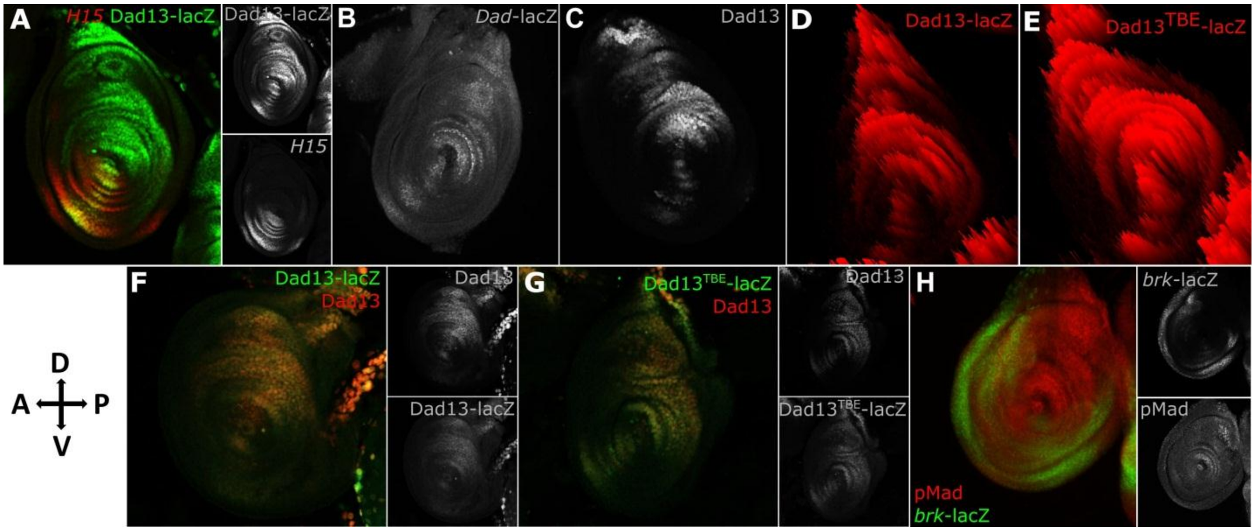

Fig. S1. Expression patterns of *Dad*, *Dad13*, *Dad13*^{TBE} and *brk.* (A) Dad13-driven expression (green, upper inset) detected with anti-β-Galactosidase antibody and H15 expression (NMR1 antibody. Red, lower inset) form a border in a third instar leg imaginal disc (n=19). H15 expression is restricted to the ventral domain and marks the expression of its redundant gene, *mid*. Dad13 is a slightly weaker read-out of Dpp signalling and like Dpp is strongly expressed in the dorsal domain and weakly expressed in the ventral domain. (B-C) Both (B) *Dad*-lacZ (n=10) and (C) Dad13-driven expression are expressed in the leg imaginal disc, with stronger expression in the dorsal domain and weaker expression the ventral domain (n=7). (C) Dad13-driven ventral expression is weaker than (B) *Dad*-lacZ. (D-E) The 2.5D processing tool in ZEN software was used to visualize intensity values of the lacZ staining for both Dad13 and Dad13^{TBE}. This tool generates a graphical read-out of signal intensity with each line representing an intensity spike. Therefore, 2.5D processing shows staining intensity. The expression of (E) Dad13^{TBE} (n=4) is more intense in both the ventral and dorsal domain compared to (D) Dad13 (n=3). (F-G) Third instar imaginal discs were stained for β-Galactosidase (green) and imaged for RFP (red). (F) Discs expressing generated Dad13 construct (Dad13-lacZ) and Dad13nRFP show nearly identical expression patterns as indicated by the merging of green and red to generate yellow. Both forms of Dad13-driven expression are weak in the ventral domain (single channel

Biology Open • Supplementary information

insets) (n=9). (G) Discs expressing generated Dad13^{TBE} construct (Dad13^{TBE}-lacZ) and Dad13nRFP show slightly different expression patterns as indicated by a stronger and wider green expression (single channel insets) (n=23). (H) pMad and *brk* form a border in the leg imaginal disc with *brk* expression (green, top inset) ventral/lateral and pMad broadly expressed in the dorsal domain and narrowly expressed in the ventral domain (red, bottom inset) (n=11). Compass indicating that discs in this report are orientated dorsal up, anterior left.

Biology Open • Supplementary information

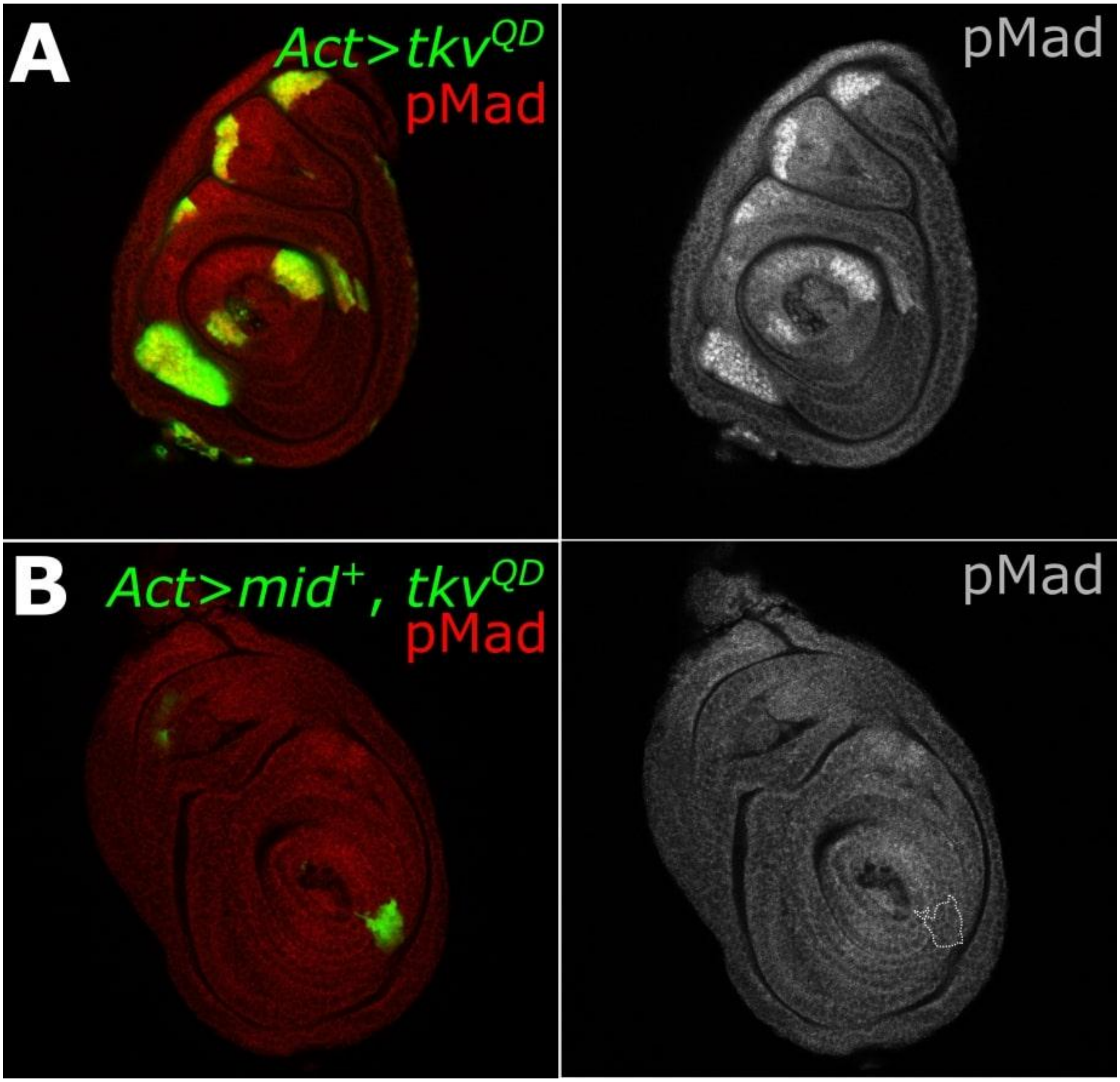

Fig. S2. pMad supressed in clones co-expressing *mid* and Dpp signaling. (A) *Ay*Gal4 gain-of-function clones expressing UAS-*tkv*^{QD} (green) results in increased pMad staining (red, single channel)(n=26). (B) When *Ay*Gal4 clones are generated which express UAS-*mid*$^{+}$ and UAS-*tkv*^{QD} (green) the pMad staining is suppressed (red, single channel, clone outlined) (n=8).

Biology Open • Supplementary information

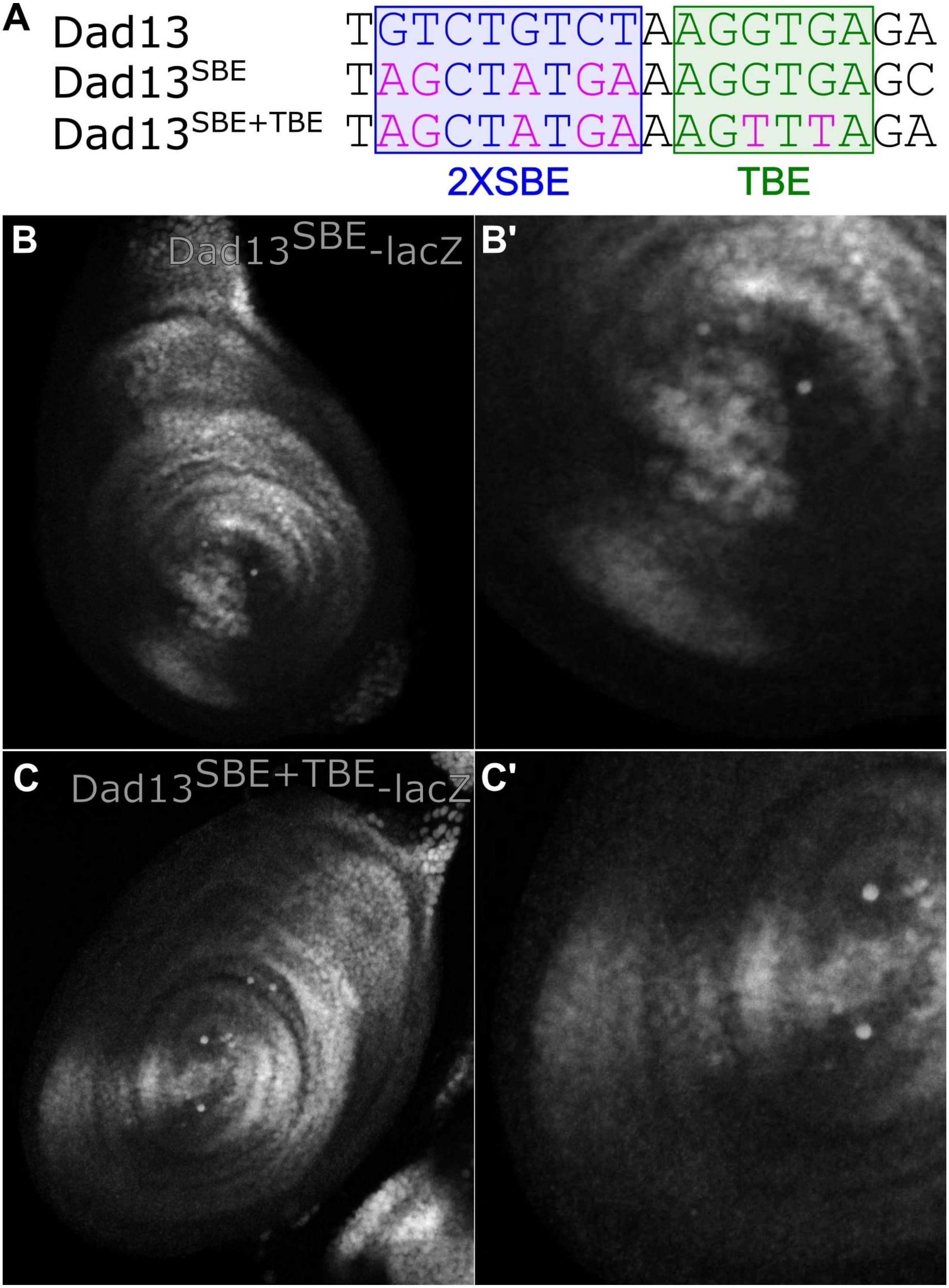

Biology Open • Supplementary information

Fig. S3. Expression patterns of other Dad13 constructs. (A) Part of the Dad13 enhancer fragment sequence, showing the 2X SBE sequence (blue) and the TBE sequence (green). Two G to T substitutions in the TBE were generated for the Dad13^{TBE} construct (fuchsia). Five nucleic acids were mutated (fuchsia) within the two SBE to generate the Dad13^{SBE} construct. A construct was also created that contained both the SBE and TBE mutations, termed Dad13$^{SBE+TBE}$. (B) Dad13^{SBE} expression is similar to Dad13 expression, as detected by lacZ with strong staining in the dorsal domain and weak staining in the ventral domain (n=25). (B') Magnified image of ventral Dad13^{SBE}-lacZ reporter expression. (C) The double mutant construct, Dad13$^{SBE+TBE}$, drives reporter expression pattern similar to Dad13^{TBE} alone, with a wider and more intense expression in the ventral domain than wild type Dad13 (n=7). Similar to Dad13^{TBE}, Dad13$^{SBE+TBE}$ dorsal expression is stronger than Dad13. (C') Magnified image of ventral Dad13$^{SBE+TBE}$-lacZ reporter expression.
